# Supplementary material for: Functional identification of soluble uric acid as an endogenous inhibitor of CD38
Source: eLife. 2024 Nov 11;13:RP96962. doi: 10.7554/eLife.96962 (PMC11554305; doi:10.7554/eLife.96962)
Supplement: Supplementary file 1. [file elife-96962-supp1.docx]

Supplementary file 1. Comparison between K_i_ values and mean levels of sUA in different tissues.

|  | Hydrolase  K_i_ (μM) | Cyclase  K_i_ (μM) | Tissue sUA (μmol/kg) |
| --- | --- | --- | --- |
| Brain | 83.5 | 71.1 | 9.8 |
| Liver | 77.3 | 76.7 | 233.4 |
| Lung | 82.5 | 77.0 | 364.6 |
| Heart | 80.6 | 70.9 | 25.2 |
| Kidney | 84.7 | 78.2 | 26.0 |
| Spleen | 70.5 | 78.1 | 83.3 |
| Ileum | 79.9 | 72.1 | 226.2 |
| Skeletal Muscle | 83.0 | 93.3 | 19.1 |

K_i_ values were also shown in Figure 1E, and tissue sUA levels were from WT mice that received 1-day treatment of saline (also shown in Figure 3-figure supplement 5A).
