# Supplementary figures and images for: Functional identification of soluble uric acid as an endogenous inhibitor of CD38
Source: eLife. 2024 Nov 11;13:RP96962. doi: 10.7554/eLife.96962 (PMC11554305; doi:10.7554/eLife.96962)

## Slide 1
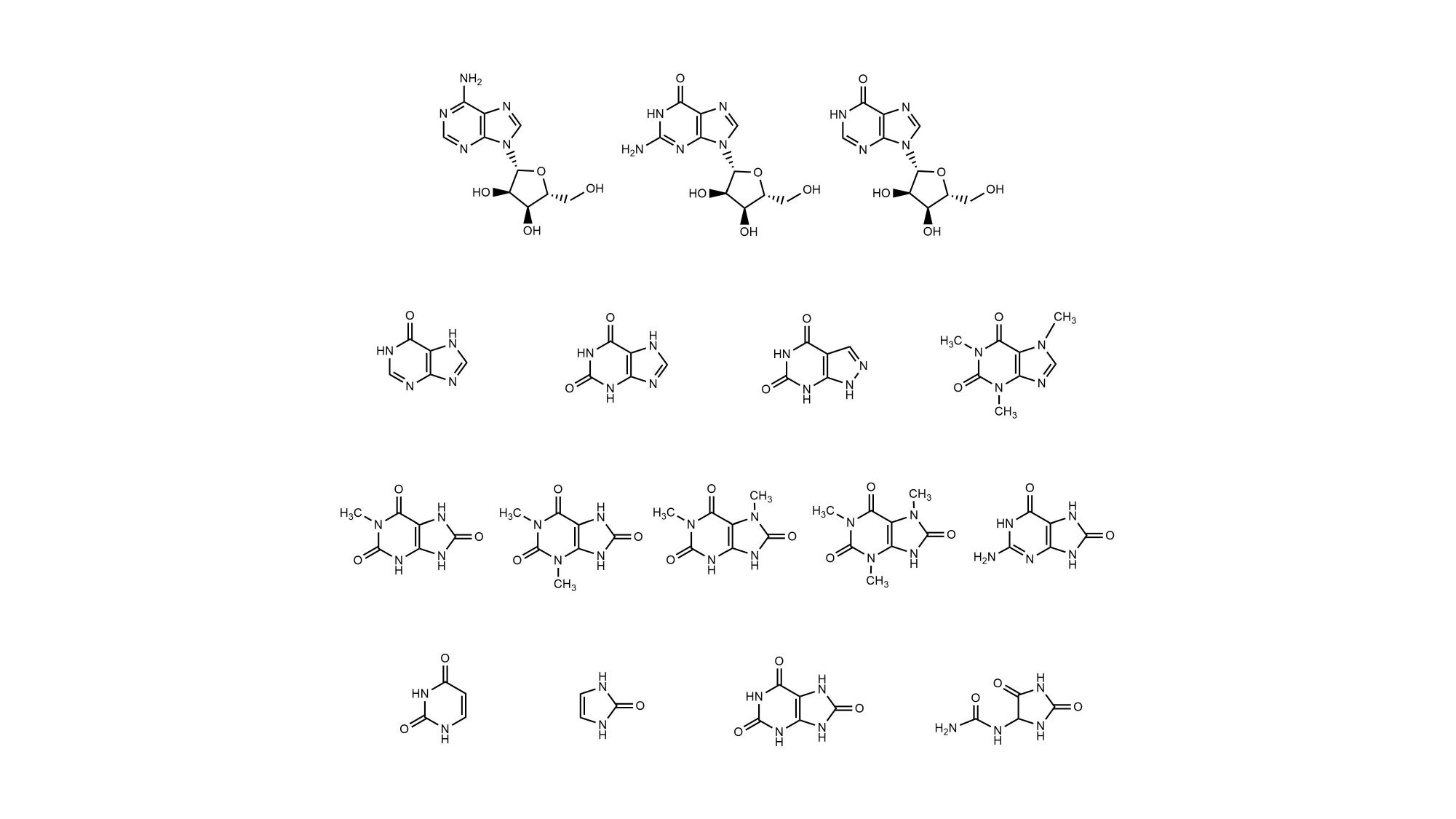

Supplement: Supplementary file 3. [file elife-96962-supp3.pptx]
